# Diabetes Mellitus Independently Predicts Survival in Beta-Lactam-Associated Hypokalemia

**DOI:** 10.14740/jocmr6253

**Published:** 2026-10-23

**Authors:** Muriel Jansen, Friedrich Stasche, Wajima Safi, Oliver Ritter, Daniel Patschan

**Affiliations:** a Department of Internal Medicine I - Cardiology, Nephrology and Internal Intensive Medicine, University Hospital Brandenburg, Brandenburg Medical School Theodor Fontane, 14770 Brandenburg an der Havel, Germany

**Keywords:** BLA, Hypokalemia, Mortality, Diabetes mellitus, COPD

## Abstract

**Background:**

Hypokalemia is a lesser-known potential side effect of any beta-lactam antibiotic (BLA) therapy. The aim of our study was to identify risk factors and outcome variables of BLA-associated hypokalemia.

**Methods:**

This was a retrospective, database-based, monocentric observational cohort study. Women and men undergoing BLA therapy due to a suspected or confirmed bacterial infection were included. Anamnestic, clinical, and laboratory variables were recorded.

**Results:**

A total of 198 patients who received BLA treatment for hypokalemia and 124 individuals without hypokalemia were included in the study. The intrahospital survival rate was significantly lower in the hypokalemia cohort. Chronic obstructive pulmonary disease (COPD) was identified as an independent predictor of death, while diabetes mellitus was identified as an independent predictor of survival.

**Conclusions:**

BLA-associated hypokalemia is associated with an elevated mortality rate and, as a result, requires prompt treatment. COPD and diabetes mellitus are each independently predictive of death and survival.

## Introduction

Hypokalemia is nearly as common as hyponatremia in inpatient settings [1]. Affected individuals are at considerable risk of cardiac arrhythmias, which can be fatal [2]. More frequent causes of nosocomial hypokalemia are the use of loop diuretics or thiazides, as well as diarrhea of variable etiology [3]. Beta-lactam antibiotics (BLAs) are an indispensable part of antimicrobial therapies for both community-acquired and hospital-acquired bacterial infections worldwide. In contrast to aminoglycosides, for example, whose use requires regular blood level monitoring to ensure therapeutic safety, the tolerability of BLAs is generally good. Better-known side effects include allergic reactions [4], possibly with kidney involvement (interstitial nephritis [5]) and effects on the intestinal microbiome (aminopenicillins) [6]. Central nervous effects have been described, but are rarely relevant in clinical practice [7]. Less well known to many physicians is the undesirable effect of renal potassium loss when using BLAs. Due to their effect as non-absorbable anions, BLAs can bind potassium cations to the tubular lumen of the kidneys, with substance-specific differences [8–10]. Cases of BLA-associated hypokalemia were first published in 1968 [11], with further reports on various substances in the BLA group appearing over the next 50 years to the present day. Recently, our group published a comprehensive review article on the topic [12]. Over the years, the spectrum of BLAs used in hospitals has changed slightly. Substances such as carbenicillin or nafcillin, for example, are now rarely used. However, also today, some patients develop hypokalemia that cannot be explained by conventional causes, even with the most commonly used preparations.

The aim of this retrospective study was to compare the characteristics of patients treated with BLAs with and without hypokalemia. In particular, risk factors and outcome characteristics of hypokalemia in BLA-treated patients were to be identified.

## Materials and Methods

### Design

This is a retrospective, monocentric observational cohort study, conducted at the Center for Internal Medicine 1 of the Brandenburg University Hospital (Brandenburg Medical School). The study was reviewed and approved by the Ethics Committee of the Brandenburg Medical School (E-01-20230321). The requirements of the Declaration of Helsinki were taken into account.

### Patients

All patients were recruited from the inpatient ward of the University Hospital. This was based on the results of the medical documentation stored in the University Hospital’s central medical database. Patients were selected if they had received at least one of the following BLAs: flucloxacillin, ampicillin (with or without sulbactam), amoxicillin, cefuroxime, ceftriaxone, ceftazidime, piperacillin/tazobactam, or meropenem. The selection process included allocation to a hypokalemia or normokalemia cohort. The decisive factor was the initial diagnosis of electrolyte disturbance after the start of beta-lactam therapy. Extensive anamnestic, clinical, and laboratory data were documented to characterize the cohorts: age, sex, hospital length of stay, body mass index (BMI), time to hypokalemia, minimum potassium concentration during the stay, potassium concentration before discharge (dead or alive), intrahospital survival, new diagnosis of cardiac arrhythmia after hypokalemia diagnosis, therapy with diuretics or diarrhea during hospital therapy, documented dehydration, serum sodium, C-reactive protein (CRP), and eGFR (initial, minimum (sodium and estimated glomerular filtration rate (eGFR))/maximum (CRP), and before discharge). Finally, comorbidities were recorded: liver cirrhosis, arterial hypertension, coronary heart disease (CHD), heart failure (HF), pre-existing atrial fibrillation, diabetes mellitus, chronic obstructive pulmonary disease (COPD), and positive neoplasia history. Tobacco inhalation was also recorded.

### Statistical analysis

Continuous variables are presented as mean ± standard deviation, or as median with range depending on their distribution; categorical variables are presented as absolute and relative frequencies. The distribution of continuous variables was assessed for normality using the Shapiro–Wilk test and visual inspection of histograms and Q–Q plots. Normally distributed continuous variables were compared between the hypokalemia and normokalemia cohorts using Student’s *t*-test for independent samples; non-normally distributed variables were compared using the Mann–Whitney U test. Categorical variables were compared using the chi-square test, or Fisher’s exact test when expected cell counts were < 5. Associations between continuous variables (e.g., length of hospital stay, minimum serum potassium concentration, BMI) were assessed using Spearman’s correlation coefficients.

Independent predictors of intrahospital mortality in the hypokalemia cohort were identified using multivariable logistic regression. Candidate covariates were selected using a two-step approach: (1) all variables that differed significantly (P < 0.05) between survivors and non-survivors on univariate analysis were considered as candidates (diabetes mellitus, COPD); (2) age, sex, diuretic therapy, and minimum eGFR were additionally included a priori as clinically established confounders/mediators of in-hospital mortality and of the potassium–outcome relationship, irrespective of their univariate significance. Because only 25 patients died during hospitalization, the number of covariates entered into each model was deliberately restricted to maintain an adequate events-per-variable ratio (approximately 4–5 events per covariate) and to reduce the risk of overfitting; diabetes mellitus and COPD were therefore each evaluated in a separate model together with the shared covariates age, sex, diuretic therapy, and minimum eGFR (model 1 and model 2), rather than in a single combined model.

Biologically plausible two-way interaction terms between covariates included in the final models (e.g., diabetes mellitus × COPD, age × sex, diuretic therapy × minimum eGFR) were tested by adding the corresponding cross-product terms to the respective model. Results of the logistic regression models are reported as odds ratios (ORs) with corresponding 95% confidence intervals (CIs). Missing data were infrequent (< 5% for all variables included in the analyses) and were handled using complete-case analysis, i.e., patients with a missing value for a given variable were excluded from that specific analysis. A two-sided P-value < 0.05 was considered to indicate statistical significance for all analyses. Statistical analyses were performed using Wizard for MacOS (version 2.0.6).

## Results

### Patients

A total of 198 hypokalemic patients (91 females, 107 males, age 73.9 ± 13.8 years) and 124 normokalemic patients (40 females, 84 males, age 69.4 ± 17.3 years) receiving BLA therapy were included in the study. The following BLAs were used in the hypokalemia cohort (descending frequency): piperacillin/tazobactam 42.4% (n = 84), ampicillin/sulbactam 25.8% (n = 51), ceftriaxone 15.2% (n = 30), flucloxacillin 7.1% (n = 14), meropenem 5.1% (n = 10), ceftazidime 2% (n = 4), cefuroxime 1.5% (n = 3), and penicillin G 1% (n = 2). Patients in the normokalemia cohort were treated as follows (in descending order of frequency): piperacillin/tazobactam 41.1% (n = 51), ampicillin/sulbactam 19.4% (n = 24), ceftriaxone 19.4% (n = 24), flucloxacillin 6.5% (n = 8), meropenem 5.6% (n = 7), cefuroxime 4.8% (n = 6), ampicillin 2.4% (n = 3), and ceftazidime 0.8% (n = 1). In the hypokalemia cohort, the time from initiation of therapy to diagnosis of electrolyte disturbance was 4 ± 2.8 days. The mean duration of antibiotic treatment in the total cohort was 9.7 ± 5.3 days. The infectious causes for the use of anti-infective agents were (in descending order of frequency): pneumonia 36.8%, urinary tract infection 14.58%, gastroenteritis 11.1%, cholecystitis 5.5%, erysipelas 2.7%, and other causes 29.32%. Table 1 summarizes all baseline characteristics of the patients in both groups.

**Table 1:** Patient Characteristics

Variable	NBLA	HBLA	P-value
Age, years (mean ± SD)	69.4 ± 1.5	73.9 ± 0.9	0.05
Gender, female (%)	32.3	46	0.015*
In-hospital therapy, days (mean ± SD)	13 ± 0.9	19.3 ± 0.8	< 0.001*
BMI, kg/m^2^ (mean ± SD)	27.2 ± 0.6	27.2 ± 0.5	0.9
In-hospital survival (%)	96	87.4	0.01*
Administration of diuretics (%)	39.5	57.6	0.002*
Diarrhea (%)	2.4	7.6	0.05
Polyuria, not induced by diuretics (%)	0	1	0.99
Dehydration (%)	18.5	21.2	0.56
Morbidities (%)			
Cirrhosis	0.8	9.6	0.001*
Arterial hypertension	78.2	82.8	0.35
CAD	21.8	22.7	0.8
HF	22.6	40.4	< 0.001*
AF	15.3	34.3	< 0.001*
COPD	17.7	19.2	0.75
DM	26.6	36.9	0.05
Obesity	75.8	73.7	0.67
History of neoplasia	23.4	27.3	0.43
Smoking (%)	91.1	86.3	0.19
Laboratory findings			
Initial serum sodium, mmol/L (mean ± SD)	136.2 ± 0.4	136.6 ± 0.4	0.36
Minimal serum sodium, mmol/L (mean ± SD)	135.5 ± 0.4	133.7 ± 0.3	< 0.001*
Serum sodium at discharge, mmol/L (mean ± SD)	139.2 ± 0.3	140.3 ± 0.4	0.19
Initial eGFR, mL/min (mean ± SD)	64 ± 2.7	60 ± 2	0.29
Minimal eGFR, mL/min (mean ± SD)	59.1 ± 2.8	49.8 ± 2.1	0.01*
eGFR at discharge, mL/min (mean ± SD)	67.6 ± 2.9	72.2 ± 2.2	0.29
Initial CRP, mg/L (mean ± SD)	85.5 ± 7.9	101.9 ± 7.7	0.41
Peak CRP, mg/L (mean ± SD)	131.1 ± 8.4	185.3 ± 7.5	< 0.001*
CRP at discharge, mg/L (mean ± SD)	38.3 ± 3.5	56.6 ± 4.2	0.01*

*P < 0.05 (statistical significance). AF: atrial fibrillation; BMI: body mass index; CAD: coronary artery disease; COPD: chronic obstructive pulmonary disease; CRP: C-reactive protein; DM: diabetes mellitus; eGFR: estimated glomerular filtration rate; HBLA: hypokalemia under beta-lactam therapy; HF: heart failure; NBLA: normokalemia under beta-lactam therapy; SD: standard deviation.

### Risk factors

A number of patient-related variables were compared to identify potential risk factors for hypokalemia associated with BLA use. This section will only mention all significant differences between the two groups. Please refer to Table 1 for the results of all analyses. There were significantly fewer women in the normokalemia group (32.2% vs. 46%, P = 0.015). Individuals with hypokalemia were treated significantly longer in the hospital (19.3 ± 0.8 vs. 13 ± 0.9 days, P < 0.001). There were also differences in various morbidities. For example, hypokalemic patients were more likely to suffer from chronic heart failure (40.4% vs. 22.6%, P < 0.001), and also more likely to suffer from atrial fibrillation (34.3% vs. 15.3%, P < 0.001) and liver cirrhosis (9.6 %vs. 0.8%, P = 0.001). Diuretics were taken more frequently by hypokalemic patients (57.6% vs. 39.5%, P = 0.002). The lowest eGFR (CKD-EPI) measured during hospitalization was lower in hypokalemic individuals (49.8 ± 2.1 vs. 59.1 ± 2.8 mL/min, P = 0.01), as was the minimum serum sodium concentration measured (133.7 ± 0.3 vs. 135.3 ± 0.4 mmol/L, P < 0.001). The maximum measured CRP and the CRP at discharge were higher in hypokalemic patients (185.2 ± 7.5 vs. 131.1 ± 8.4 mg/L, P < 0.001 and 56.6 ± 4.2 vs. 38.3 ± 3.5 mg/L, P = 0.003).

### Outcome

#### Survival

The survival rate was significantly lower in hypokalemic patients (87.4% vs. 96%, P = 0.01). In the hypokalemia cohort, some covariates were associated with patient survival. At the time of hospital discharge (dead or alive), CRP was significantly higher in the deceased (129.8 ± 17.6 vs. 45.3 ± 3.3 mg/L, P < 0.001). In addition, the minimum eGFR measured during in-hospital therapy was lower in non-survivors than in survivors (37.7 ± 5.7 vs. 51.5 ± 2.2 mL/min, P = 0.019). Finally, several morbidities were associated with in-hospital mortality: diabetes mellitus was more common among survivors (40.5% vs. 12%, P = 0.006), while COPD was more common among non-survivors (36% vs. 16.8%, P = 0.02). More non-survivors had a history of neoplasia (48% vs. 24.8%, P = 0.013) (Table 2). The two covariates, diabetes mellitus and COPD, were finally included in a multiple logistic regression analysis, in combination with other covariates (age, gender, diuretics, minimal eGFR), with intrahospital death as the dependent variable. Both diabetes mellitus and COPD were independent predictors of death (model 1, COPD, OR 3.3 (1.2–8.6)) and survival (model 2, no diabetes mellitus, OR 4.8 (1.5–21.1)), respectively. Table 3 and Figure 1 summarize the results from all survival analyses, including the logistic regression (Table 3).

**Table 2:** All Variables Compared Between Surviving and Deceased Patients With Hypokalemia on Beta-Lactam Therapy

Variable	Survival	Death	P-value
Age, years (mean ± SD)	73.4 ± 1	77.4 ± 2.1	0.27
Gender, female (%)	46.8	40	0.52
in-hospital therapy, days (mean ± SD)	19.6 ± 0.9	17.2 ± 1.5	0.73
BMI, kg/m^2^ (mean ± SD)	27.4 ± 0.6	26 ± 1.3	0.39
Administration of diuretics (%)	56	57.8	0.86
Diarrhea (%)	8.7	0	0.12
Polyuria, not induced by diuretics (%)	1.2	0	0.58
Dehydration (%)	21.4	20	0.87
Morbidities (%)			
Cirrhosis	8.7	16	0.24
Arterial hypertension	83.2	80	0.68
CAD	20.8	36	0.09
HF	42.2	28	0.17
AF	34.1	36	0.85
COPD	16.8	36	0.02*
DM	40.5	12	0.006*
Obesity	73.4	76	0.78
History of neoplasia	24.3	48	0.013*
Smoking (%)	13.4	16	0.72
Laboratory findings			
Minimal serum potassium, mmol/L (mean ± SD)	3.14 ± 0.02	3.16 ± 0.05	0.65
Initial serum sodium, mmol/L (mean ± SD)	136.5 ± 0.4	137.3 ± 1.2	0.53
Minimal serum sodium, mmol/L (mean ± SD)	133.8 ± 0.4	133.1 ± 1	0.34
Serum sodium at discharge, mmol/L (mean ± SD)	139.8 ± 2.2	143.7 ± 2.2	0.36
Initial eGFR, mL/min (mean ± SD)	61.2 ± 2.2	51.4 ± 5.5	0.14
Minimal eGFR, mL/min (mean ± SD)	51.5 ± 2.2	37.7 ± 5.7	0.019*
eGFR at discharge, mL/min (mean ± SD)	73.1 ± 2.3	66.8 ± 7.3	0.51
Initial CRP, mg/L (mean ± SD)	98.9 ± 8	122 ± 25	0.42
Peak CRP, mg/L (mean ± SD)	179.8 ± 7.8	222.7 ± 23.4	0.06
CRP at discharge, mg/L (mean ± SD)	45.4 ± 3.3	129.8 ± 17.6	< 0.001*

*P < 0.05 (statistical significance). A total of five significant differences between the two cohorts were identified, and four variables were included in the logistic regression analysis. AF: atrial fibrillation; BMI: body mass index; CAD: coronary artery disease; COPD: chronic obstructive pulmonary disease; CRP: C-reactive protein; DM: diabetes mellitus; eGFR: estimated glomerular filtration rate; HF: heart failure; SD: standard deviation.

**Table 3:** Results of the Logistic Regression Analysis

Variable	Odds ratio	95% CI
Model 1: endpoint – survival; variables - age, gender, diuretics in use, minimal eGFR, COPD		
Age	1.017	0.9803 to 1.059
Female gender	0.5758	0.2213 to 1.424
Diuretics	0.5811	0.2288 to 1.481
Minimal eGFR	0.9784	0.9598 to 0.9960
COPD	3.318	1.246 to 8.605
Model 2: endpoint – survival; variables - age, gender, diuretics in use, minimal eGFR, diabetes mellitus		
Age	1.015	0.9776 to 1.058
Female gender	0.5825	0.2256 to 1.434
Diuretics	0.6105	0.2421 to 1.549
Minimal eGFR	0.9803	0.9609 to 0.9986
(no) diabetes mellitus	4.805	1.556 to 21.11

Two models were used, the only difference between the models was the inclusion of either COPD (model 1) or diabetes mellitus (model 2) as an independent variable. In both models, age, sex, use of diuretics, and the minimum eGFR during inpatient treatment were defined as additional independent variables. CI: confidence interval; COPD: chronic obstructive pulmonary disease; eGFR: estimated glomerular filtration rate.

**Figure 1:**
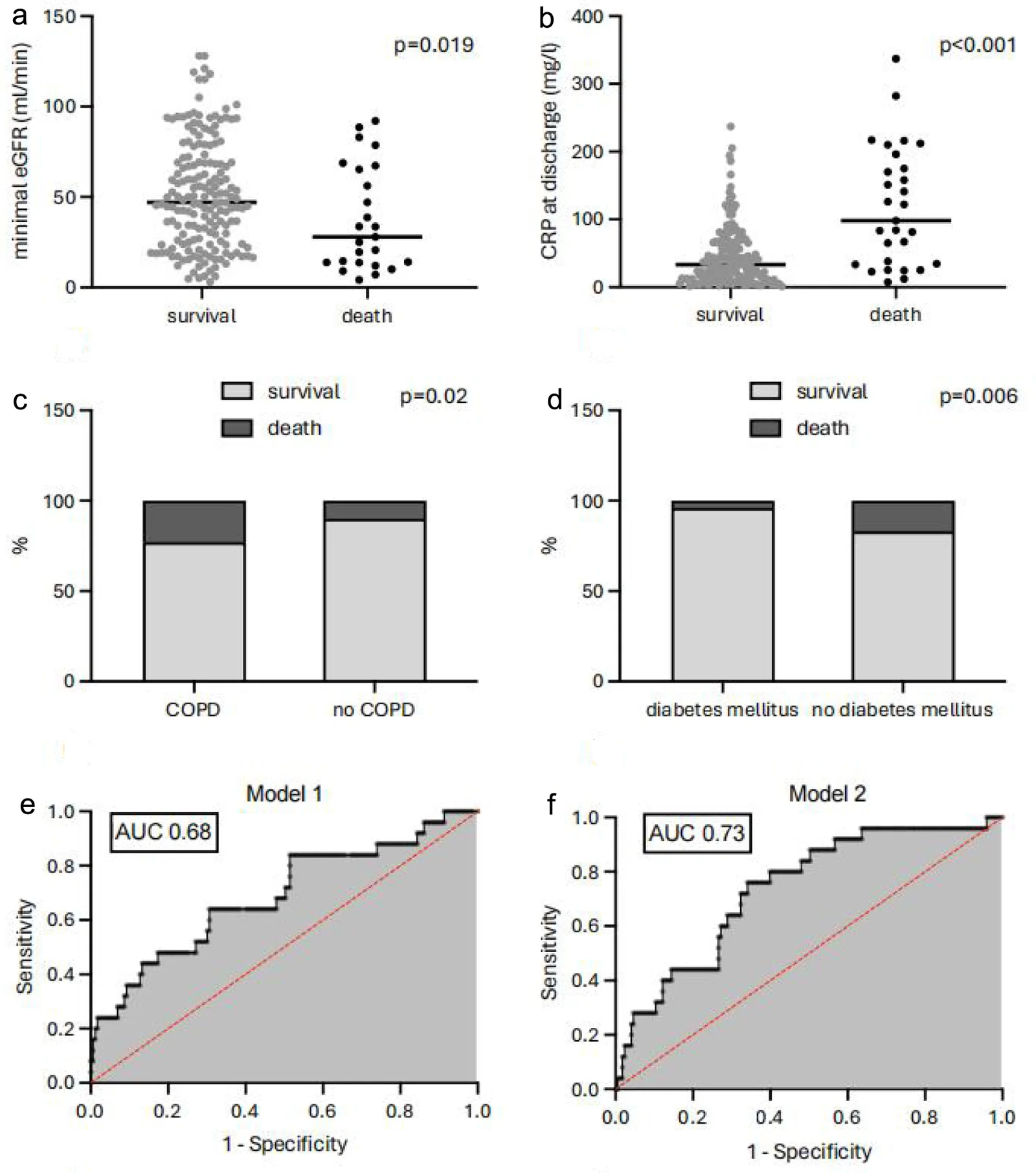
Key results of the survival analysis. Deceased individuals had a lower minimum eGFR (a), a higher peak CRP (b), and suffered more from COPD (c) but less from diabetes mellitus (d). (e, f) AUC of the two logistic regression models.

#### Duration of in-hospital therapy

The length of hospital stay was positively correlated with the time between the start of BLA therapy and the diagnosis of hypokalemia (P = 0.015), and there was also a negative correlation with the minimum potassium concentration (P = 0.02). The longer the hypokalemic patients stayed in hospital, the lower the serum sodium concentration at discharge (dead or alive) (P = 0.015). Finally, the length of hospital stay was longer in hypokalemic patients with a positive history for neoplasia (23 ± 1.8 vs. 17.9 ± 0.9 days, P = 0.008).

#### Minimal serum potassium

In hypokalemic individuals, the minimum serum potassium concentration correlated positively with the BMI (P = 0.02). It was also lower in hypokalemic individuals with a positive history of neoplasia (3 ± 0.04 vs. 3.1 ± 0.02 mmol/L, P = 0.001).

### Subgroup analysis of diabetic patients

Of all diabetic patients, four had type 1 diabetes mellitus; all others had been diagnosed with type 2 diabetes mellitus. The following variables were compared between diabetic and non-diabetic patients: age, gender, in-hospital treatment time, BMI, BLA therapy duration until diagnosis of hypokalemia, serum potassium (minimum and before discharge), incidence of cardiac arrhythmias after diagnosis of hypokalemia, diuretic therapy, diarrhea , dehydration, polyuria (not diuretic-induced), serum sodium, CRP, and eGFR (each at three time points), as well as the comorbidities liver cirrhosis, heart failure, atrial fibrillation, COPD, and obesity. No variable differed between diabetics and non-diabetics. Diabetic individuals merely had a more frequent positive history of tumor diseases (35.6% vs. 22.4 %, P = 0.04).

## Discussion

This study analyzed a medical issue often overlooked in patients taking BLAs: low potassium levels. Beta-lactams potentially cause hypokalemia by creating a negative gradient on the inner side of the collecting duct, which leads to potassium being excreted by the kidneys [11, 13]. This effect is primarily attributable to the electrophysiological property of beta-lactams as non-reabsorbable anions; these are class-specific drug actions. Another mechanism may be enhanced sodium delivery: an electronegative luminal gradient draws Na^+^ into the principal cells of the collecting duct, stimulating the Na^+^/K^+^-ATPase and thereby increasing potassium wasting [14]. Finally, diuretic-induced metabolic alkalosis contributes to hypokalemia.

The incidences of BLA-associated hypokalemia reported in the literature vary widely, not least depending on the substances used. In a prospective study published in 2021, the electrolyte imbalance was reported in about 15% of patients receiving meropenem therapy [15]. Two years earlier, however, hypokalemia was found in 42% of all patients treated with flucloxacillin. In contrast, the incidence was 14% when ceftriaxone was used [16]. Our study was not designed to identify the frequency of hypokalemia during BLA therapy; rather, the aim was to identify risk factors and the outcome of those affected.

Our findings are surprising. Some differences between normokalemic and hypokalemic patients under BLA therapy could be identified. Individuals with hypokalemia stayed in hospital longer, received diuretics more often, and suffered more frequently from liver cirrhosis, cardiac insufficiency, and atrial fibrillation. In addition, the mean CRP was higher both maximally and before discharge from hospital than in normokalemic patients. All of these factors can be seen as indicators of increased morbidity. It may even be possible to assume gender-related risk differences, with more women being represented in the hypokalemia cohort. The fact that hypokalemic patients were generally sicker is also evident in their survival rates, which were significantly lower. Hypokalemic patients who died had worse kidney function and higher CRP levels than survivors. Our study did not distinguish between acute and chronic kidney disease. Reduced kidney function is nevertheless linked to higher mortality, regardless of whether it is acute or chronic [17–19]. Another key difference was the significantly higher prevalence of COPD in deceased patients. COPD as a variable even proved to be independently predictive of death during the hospital stay. This finding is not necessarily surprising, as the prognosis of COPD patients with advanced disease is comparably reduced as the prognosis of patients with advanced tumor diseases [20]. The identification of diabetes mellitus as an independent predictor of survival in contrast is quite unusual. The absence of diabetic disease was associated with an OR of over 4 for death. First of all, it should be noted that the results are probably not the consequence of a bias effect, e.g., due to the inclusion of an insufficient number of diabetic patients. Of the 198 patients with low potassium, 36.9% (n = 73) had diabetes. In the final subgroup analysis of the diabetes cohort, there were no differences compared to non-diabetics for almost all variables. It is hardly necessary to recapitulate the numerous detrimental effects of a chronically hyperglycemic metabolic state, neither in the short nor medium to long term. The prognostically unfavorable effects of diabetes not only compromise the function and structure of the blood vessel system but are also known to affect the nervous system and kidneys. Renal complications of diabetes are by no means limited to the vasculature. Regardless of the numerous detrimental cardiovascular effects of diabetes, a myriad of reports have been published on negative associations with the prognosis of orthopedic, traumatologic, oncologic, dermatologic, and numerous other diseases. At this point, it is not possible to even attempt to list these references. Nevertheless, not all effects of diabetes mellitus can be consistently traced in, for example, all age groups or even in relation to gender. In late 2023, Matsushita and colleagues published a paper on the prognosis of patients with myocardial infarction. Diabetes mellitus was identified as an independent mortality predictor in individuals under 65 years of age; beyond the age of 65, this effect did not exist. Comparable observations were made in both women and men. Only in the male collective was diabetes associated with increased mortality [21]. No satisfactory explanation was found. Inconsistent or “paradoxical” effects of diabetes mellitus have been reported repeatedly in the literature. The best-documented association is that between diabetes mellitus and abdominal aortic aneurysm (AAA). People with diabetes have a lower risk of developing an AAA in the first place, and if an aneurysm is already present, it grows more slowly—studies have reported reductions in the growth rate ranging from about 35% to 65% per month. Perioperative mortality following surgery for a ruptured aneurysm also appears to be lower in people with diabetes. The mechanisms under discussion include: increased levels of advanced glycation end products (AGEs), leading to a stiffer aortic wall that is less susceptible to dilation; reduced activity of matrix metalloproteinases (particularly MMP-2 and MMP-9); and possible independent effects of antidiabetic medications (particularly metformin) unrelated to the blood glucose-lowering effect itself. Similar, albeit weaker, evidence exists for intracranial aneurysms [22]. The “diabetes paradox” in critically ill patients describes observations that pre-existing diabetes in cases of sepsis or other acute illnesses is not consistently associated with increased mortality—and in some cases is even associated with lower mortality—in contrast to acute stress hyperglycemia in non-diabetics, which represents a clear risk factor. Chronic hyperglycemia may induce cellular adaptations that provide some protection against the consequences of an acute hyperglycemic episode [23]. Our understanding of specific prognostic outcome variables in diabetes mellitus presumably requires some revision in any case. Recent studies on cardiac involvement in diabetes, for example, have revealed an association between increased mortality and a higher-than-normal (supra-normal) left ventricular ejection fraction [24]. Another relatively recent study found that higher values on the so-called CONUT score (which provides insight into nutritional status) were independently predictive of death in patients with heart failure with preserved ejection fraction (HFpEF) [25]. It is also possible that the association between diabetes mellitus and a reduced risk of death observed in our study is linked to nutritional status variables that cannot be captured by our dataset. Another factor worth discussing is patient age. Elderly patients often have reduced renal reserve, impaired metabolic compensation, and multiple comorbidities that may increase overall susceptibility to electrolyte disturbances during antibiotic treatment. The influence of age on pH and, consequently, potassium homeostasis is discussed very convincingly, for example, in a recent narrative review [26]. However, there was no significant age difference between hypokalemic patients with diabetes who survived and those who died. Finally, the effects of antidiabetic therapies must be taken into account. In the presence of copper, metformin exerts antiproliferative and possibly antineoplastic effects [27]. On the other hand, metformin–copper interactions have also been described as harmful to the kidneys, particularly in the context of interactions with amyloid polypeptides [28]. However, it is not possible to draw any conclusions regarding the effects of antidiabetic therapies on the survival of individuals with BLA-associated hypokalemia because, as previously mentioned, diabetes therapy was not systematically documented in our study.

The elucidation of the findings of our study, and the elucidation of the underlying mechanisms, presents us with a particularly challenging task. Given that hypokalemia was associated with mortality during BLA therapy, it would be reasonable to hypothesize that the electrolyte imbalance may have exerted an influence on the outcome. It can be postulated that patients with diabetes may be more resilient to disturbances in potassium homeostasis than those without the disease. It is evident that insulin treatment can result in the development of hypokalemia, which frequently remains undiagnosed. Nevertheless, while diabetics may be more likely to tolerate hypokalemia, this does not account for the markedly life-prolonging effects of the disease in the context of BLA-associated hypokalemia. In conclusion, the question remains unanswered at the moment.

### Limitations

The retrospective design is a limitation. The same may apply to the unequal group sizes. In addition, information on renal potassium loss under BLA therapy would be desirable. However, the latter are simply not routinely available. A wide variety of beta-lactam regimens were administered and documented; concomitant medications and the morbidity profile varied significantly among patients. Variables related to nutritional status, which can have a major impact on acid-base and potassium homeostasis, were scarcely recorded. Another limitation—one that only became apparent once the results were known—is the lack of more detailed measures related to diabetes mellitus. Since the disease was identified as a predictor of survival, a number of additional variables would naturally be of interest: duration of the disease, HbA1c, medication/insulin therapy, diabetic complications, and others. These variables should have been collected at the outset. In that case, however, variables regarding other—in fact, all—comorbidities would also have had to be documented. The scope of the dataset, and thus of the article, would presumably have been too large. Nevertheless, this remains a limitation.

### Conclusions

BLA-associated hypokalemia has been demonstrated to reduce the probability of survival of patients undergoing treatment. It is therefore imperative to ensure that therapeutic control is maintained. COPD has been identified as a risk factor for mortality in this situation, whereas diabetes mellitus appears to exert a protective effect. The underlying mechanisms remain unclear.

## Data Availability

The data that support the findings of this study are not publicly available, since it contains information that could compromise the privacy of research participants but are available from daniel.patschan@mhb-fontane.de upon reasonable request.
